# Parametric Modeling of Visual Search Efficiency in Real Scenes

**DOI:** 10.1371/journal.pone.0128545

**Published:** 2015-06-01

**Authors:** Xing Zhang, Qingquan Li, Qin Zou, Zhixiang Fang, Baoding Zhou

**Affiliations:** 1 College of Information Engineering, Shenzhen University, Shenzhen, P.R. China; 2 Shenzhen Key Laboratory of Spatial Smart Sensing and Services, Shenzhen University, Shenzhen, P.R. China; 3 Key Laboratory for Geo-Environment Monitoring of Coastal Zone of the National Administration of Surveying, Mapping and GeoInformation, Shenzhen University, Shenzhen, P.R. China; 4 State Key Laboratory of Information Engineering in Surveying, Mapping, and Remote Sensing, Wuhan University, Wuhan, P.R. China; 5 School of Computer Science, Wuhan University, P.R. China; Centre de Neuroscience Cognitive, FRANCE

## Abstract

How should the efficiency of searching for real objects in real scenes be measured? Traditionally, when searching for artificial targets, e.g., letters or rectangles, among distractors, efficiency is measured by a reaction time (RT) × Set Size function. However, it is not clear whether the set size of real scenes is as effective a parameter for measuring search efficiency as the set size of artificial scenes. The present study investigated search efficiency in real scenes based on a combination of low-level features, e.g., visible size and target-flanker separation factors, and high-level features, e.g., category effect and target template. Visible size refers to the pixel number of visible parts of an object in a scene, whereas separation is defined as the sum of the flank distances from a target to the nearest distractors. During the experiment, observers searched for targets in various urban scenes, using pictures as the target templates. The results indicated that the effect of the set size in real scenes decreased according to the variances of other factors, e.g., visible size and separation. Increasing visible size and separation factors increased search efficiency. Based on these results, an RT × Visible Size × Separation function was proposed. These results suggest that the proposed function is a practicable predictor of search efficiency in real scenes.

## Introduction

For humans, one important skill is the ability to search for and visually identify target objects among irrelevant local distractions in real-world scenes (for example, searching for a hotel in the street, a book on bookshelves, or a vehicle in a parking lot). Owing to the complexity of real scenes and limited neural resources, visual search efficiency relies on a selection mechanism known as visual attention [1,2], which enables humans to allocate more neural resources to extracting the most important information from the physical environment. Visual search, in which observers intend to search for a pre-defined target among irrelevant distractors, is one of the most important paradigms for studying visual attention [3,4]. Extensive studies on visual search have greatly improved our understanding of the mechanisms of attention deployment. Most visual search studies have used simple items isolated on blank artificial backgrounds as search targets [4–6]. However, attention selection in real scenes may use partly different mechanisms from that in artificial scenes [7]. Studies performed with real scenes may better reveal the capabilities and characteristics of the human visual system [8]. Furthermore, how observers find real objects in real scenes is more interesting [9–12]. Numerous difficulties exist when constructing models of visual search in real scenes. Although natural pictures or human faces have been used as search targets in some studies [13–15], these stimuli are still isolated objects.

One difficulty with the construction of models in real scenes concerns the notion of set size [11]. The efficiency of visual search is primarily dependent on the set size, and it has been reported that there is a linear increase in response time (RT) as the number of distractors increases [16]. The slope of the RT × Set Size function describes the increased RT of adding an item (target or distractor) to the search scene. However, defining the set size is difficult in real scenes [11,17] because it is hard to count the number of objects of various categories in complex scenes. Wolfe et al. hand-labeled 100 indoor scenes and used the number of labeled regions as a surrogate for set size [10]. Their study revealed that visual search is very efficient in real scenes. However, the authors also found that set size had limited use as a predictor of RT in real scenes. The variance of other factors, such as size and color, might affect the influence of set size. It is unclear whether the RT × Set Size function is a suitable predictor of search efficiency in real scenes. How to quantitatively measure efficiency during real search tasks remains unknown.

Another factor that can affect search efficiency is a ubiquitous phenomenon known as visual crowding, contour interaction or spatial interaction [18–22]. Crowding occurs when the ability of the visual search is better when a target is presented alone than when the target is flanked by other objects in its vicinity [18,21,23,24]. Crowding has been reported in a variety of spatial tasks, including letter identification [21,25,26], Vernier acuity [27], orientation discrimination [28], stereoacuity [29], and face recognition [30–32]. Crowding represents an essential bottleneck for object perception and can impair the ability to recognize objects in clutter [18]. However, although the phenomenon of visual crowding is ubiquitous in everyday search tasks, few studies have quantitatively examined the role of crowding in real scenes [33]. How and to what extent visual crowding affects the search efficiency of real objects remains unclear.

Recently, many computational models based on bottom-up activation mechanisms have been proposed to study the mechanisms of attention guidance in real scenes [34–37]. For example, Itti and Koch proposed the saliency model [34,35], in which a task-independent visual feature (in terms of bottom-up saliency) can be computed from various low-level features, including color, intensity, and orientation. Bottom-up saliency can predict attractive regions in natural scenes. It delivers good results in situations with little contextual or top-down information during a search task but fails in real-world search tasks where context plays an important role [38]. Recent eye movement studies suggested that top-down information, such as scene context [39–41] or target templates [42,43], facilitates real-world visual search tasks because this type of information guides attention to regions with a high probability of containing the target (e.g., in real scenes, buildings are typically on the ground, not in the sky). Eye movements play an important role during the searching of complex, real-world scenes and provide a rich dataset to improve our understanding of visual search [9]. Although those studies are exploring the role of high-level knowledge in attention guidance, they also contribute quantitative measurements of search efficiency in real scenes. For example, spatial knowledge of object categories in urban scenes plays an important role in everyday search tasks [44,45]. When searching for a target building, people may pay little attention to objects from other categories, such as vegetation, vehicles or pedestrians. The target template (e.g., picture cues) can provide unique appearance information of a target and distinguish it from similar distractors.

**Fig 1:**
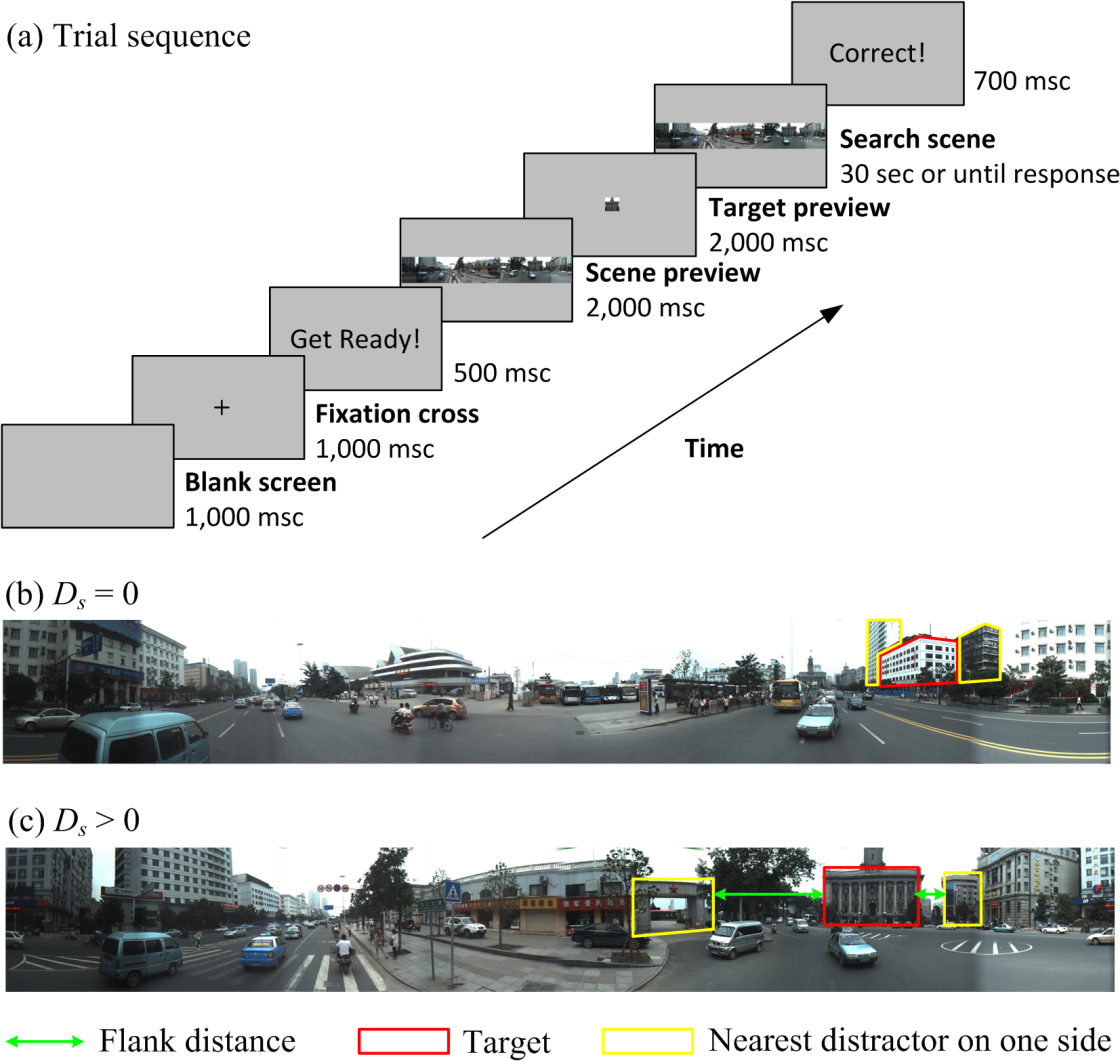
Trial procedure and scene examples for the experiment. (a) Trial sequence of the visual search paradigm. (b) An example of a search scene where the target (red polygon) is closely flanked by two distractors (green polygons) on both sides. (c) Another example of a search scene where the distractor (left) is relatively far from the target compared with another distractor (right). The scene photographs were taken manually by the author (XZ).

**Fig 2:**
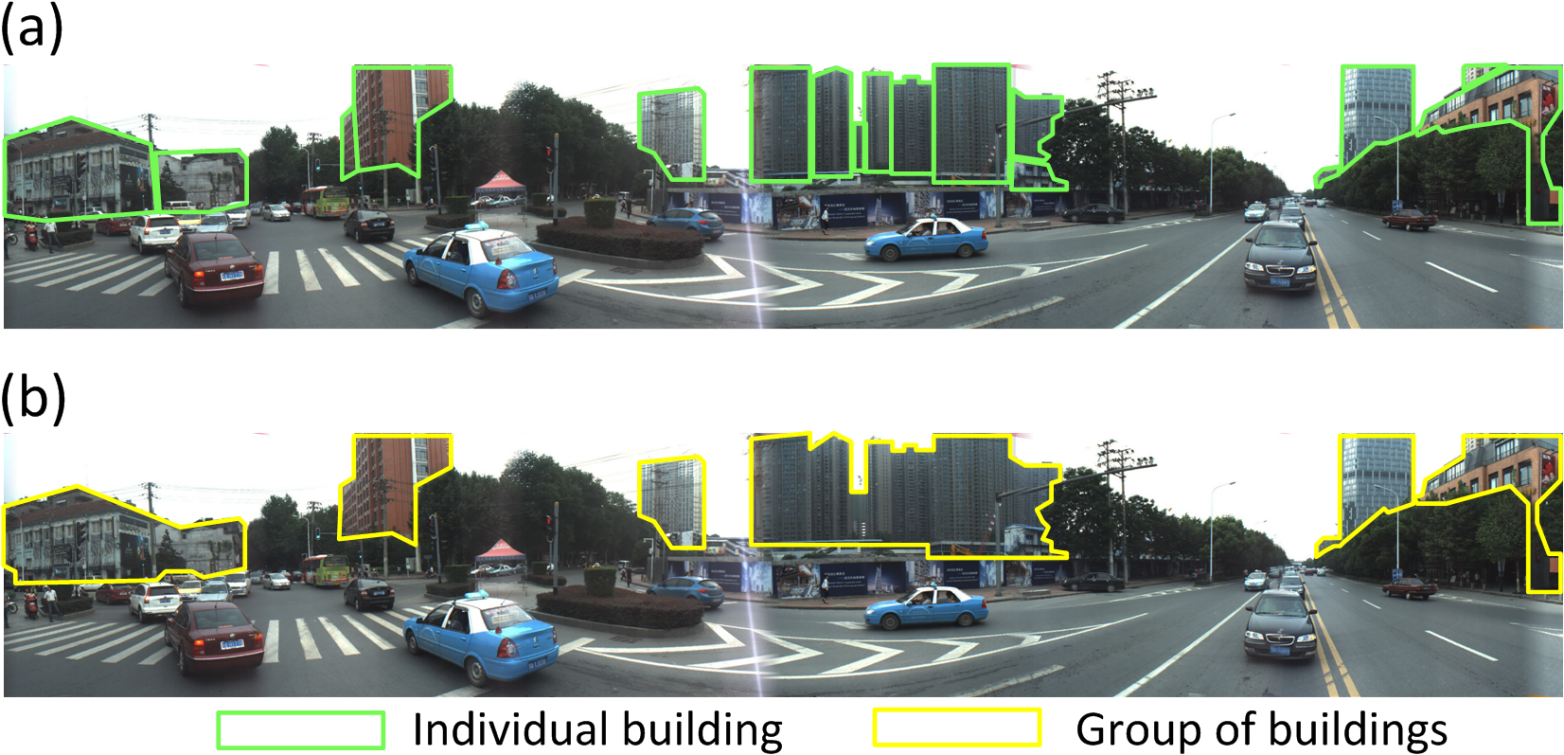
Examples of labeled objects and groups in a search scene. (a) labeled individual buildings that were counted in the set size. (b) labeled building groups formed by the individual buildings in (a).

The present study investigated the quantitative measures of search efficiency in real scenes based on a combination of low-level features and high-level features. We studied the quantitative role of several factors, including set size, grouping, visible size, visual crowding, and eccentricity, in visual search efficiency and to what extent search efficiency can be quantitatively measured by the integration of these factors. We unified the categories of target objects (buildings) and search scenes (urban scenes) for all stimuli to equalize the influence of category factor on search efficiency. We also controlled the variance of bottom-up salience by approximately equalizing the salience conditions for all targets. Pictures of target objects were used as target templates because they distinguish targets from highly heterogeneous distractors. Visible size was selected because it can reflect the amount of information about an object’s unique appearance as represented by the target picture: it was defined as the pixel number of a visible part of an object in a scene (note that any obstructed portion of an object was not included in its visible size). The influence of visual crowding was also considered. A variable, termed *D*_*s*_, described the target-flanker separation in real scenes. *D*_*s*_ refers to the sum of flank distances from a target to the nearest distractors that are in the same category as the target, in two directions (left and right). The effect of visual crowding increases with increasing similarity between the target and distractors [18], which is primarily determined by the object categories. As shown in Fig 1, the target building (red polygon) was flanked by distractors (green polygons) on both sides. The *D*_*s*_ of the target in Fig 1B was lower than that of the target in Fig 1C. The influence of grouping was also considered because recent studies have demonstrated that objects in regular configurations can be grouped to reduce the effective number of objects competing for neural representation and cognitive processing resources [7]. As shown in Fig 2, spatially adjacent objects may be grouped with each other in real scenes. We tested the influence of grouping on real-scene visual search.

We focused on three questions in the present study: (1) the quantitative contribution of multiple factors to search efficiency in real scenes; (2) whether *D*_*s*_ is a suitable descriptor of crowding in real scenes; (3) whether the RT for real objects can be quantitatively measured by a combination of low-level and high-level features.

## Methods

### Ethics statement

The experimental protocol was approved by the Institutional Review Board of Shenzhen University. The experiments and data analyses were performed in accordance with the approved guidelines and regulations. The individuals who participate in this study gave written informed consent to publish these case details.

### Observers

A group of 14 observers was tested in the experiment (3 females and 11 males; 23–30 years). The observers were students at Shenzhen University. All of the students had normal or corrected-to-normal vision. The experimental procedure was carefully explained to the students before the experiment.

### Materials

Two sets of full-color images of real scenes were used as stimuli (see Fig 1B and 1C for examples). There were 182 scene images in dataset 1 and 126 scene images in dataset 2. When displayed on the screen, each scene image covered 34.9° × 12.1° of visual angle. All images in dataset 1 were urban scenes in the city of Wuhan, and the images in dataset 2 were urban scenes in the city of Manhattan. For each scene, a target image containing a building object was copied from the scene image. When displayed on the screen, each target image was presented in the center to scale with its original size. To approximately equalize the conditions of bottom-up salience for all stimuli, the open software iLab Saliency Toolbox 2.2 (http://www.saliencytoolbox.net/index.html) developed by Walther and Koch [46] was used to calculate the salience value. The visual salience of all targets in the two datasets was less than 0.3 (the maximum was 1). In a given trial, the scene and target images were displayed on a 19-inch LCD monitor (resolution = 1,280 × 1,024; refresh rate = 60 Hz). The search results (i.e., clicking location and time) were collected automatically from observers using a hand-held mouse. The mouse pointer started in the center of the screen in all trials.

### Procedure

The participants were seated 54 cm away from the screen and were instructed to search for a target in the scene. On each trial, a gray, blank screen was first present for one second followed by a fixation cross at the center of the screen for two seconds with the words “Get ready!” for 500 msec (please see Fig 1A)). Then, the scene image was presented in the center for 2 sec followed by the target image in the center for 2 sec. After this preview, the search scene was presented in the center of the screen again, and the participants were asked to locate the target. When the participants believed that they had found the correct target, they clicked it in the search scene. The search ended when the target was clicked or after 30 seconds. The click positions and response times were recorded automatically. Between trials, information about the observer’s performance in previous searches appeared on the screen to encourage observers to perform better in the next trial. The observers performed three practice trials before performing 182 separate trials in dataset 1. All trials were presented in a random order. The 126 trials in dataset 2 were performed on a different day, following the same procedure as for 1

### Data analysis

Following the labeling method of Wolfe et al. [10], we hand-labeled all of the scene images in each dataset and counted the number of labeled objects in each scene to test the effect of set size on visual search in real scenes. Owing to the context being urban scenes, buildings were the main objects counted in the set size (see Fig 2A). Vehicles and plants were not counted because the search targets were restricted to buildings. The “road”, “ground” and “sky” were also not labeled because it would be incorrect to count them as individual objects. In addition to set size, grouping is also relevant to the efficiency of real-scene search. A recent study suggested that objects in regular configurations are grouped to reduce the effective number of objects competing for attention, contributing to the efficiency of real-scene perception [7]. We investigated whether the grouping of distractors in a search scene increased the efficiency of target detection. As shown in Fig 2B, we counted the number of building groups in each scene image. If several distractor objects (buildings) were spatially adjacent and shared contours with each other, they were counted as a group.

In the analysis, several variables were calculated to test their influence on search efficiency, including *size*, *D*_*s*_, *eccentricity* and *relative size*. The target *size* was defined as the pixel number of its visible portion in the scene image. *D*_*s*_, which represents target-flanker separation, was calculated as the sum of flank distances from a target to the nearest distractors in two directions (please see Fig 1B and 1C for examples). *Eccentricity* represents the distance from the center of a target to the fixation cross on the screen. The *relative size* is a measure of the size difference between a target and the distractors in a scene, which was calculated as follows:

$$relative\;size=\frac{n\cdot size}{\sum_{i=0}^{n}size\prime \;\left(i\right)}$$ (1)

where *size* is the visible size of a target object; n is the number of distractor objects in the scene; and *size*’(*i*) is the *size* of the *i*th distractor. The *size* attribute of the distractors was calculated according to the labeling results of the search scenes, as shown in Fig 2A.

An analysis of covariance (ANCOVA) was performed to investigate the effect of different factors, including set size, grouping, visible size (or relative size), separation and eccentricity, on the search efficiency. These factors were used as continuous independent variables in the ANCOVA. To linearize the relation between RT and each variable, we log-transformed the RT and each independent variable (*set size*, the number of groups, *size*, *relative size*, *D*_*s*_ and *eccentricity*). Subject was used as a random categorical variable.

To study the influence of visible size during conditions when targets were closely flanked by distractors on both sides (see Fig 1B for example), log-transformed RT was plotted as a function of *size*. A linear function was used to fit the data:

logRT′=a⋅logsize+d (2)

where *RT'* is the estimation of the RT for a target object; *size* is the visible size of the target; *a* and *d* are constants.

To further model the effect of visible size and separation, log-transformed RT was plotted as a function of both *size* and *D*_*s*_. Eq (2) was extended as follows:

$$\text{log}\;RT\prime =a\cdot \text{log}\;size+b\cdot \text{log}\;D_{s}+d$$ (3)

where *RT'* is the estimation of the RT for a target object; *size* is the visible size of the target; *D*_*s*_ is the sum of the flank distance; *a*, *b* and *d* are constants.

When *eccentricity* was included as a variable in the function, Eq (3) was extended as follows:

$$\text{log}\;RT\prime =a\cdot \text{log}\;size+b\cdot \text{log}\;D_{s}+c\cdot \text{log}\;ecc+d$$ (4)

where *RT*' is the estimation of the RT for a target object; *size* is the visible size of the target; *D*_*s*_ is the sum of the flank distance; *ecc* is the target eccentricity; *a*, *b*, *c* and *d* are constants.

## Results

**Fig 3:**
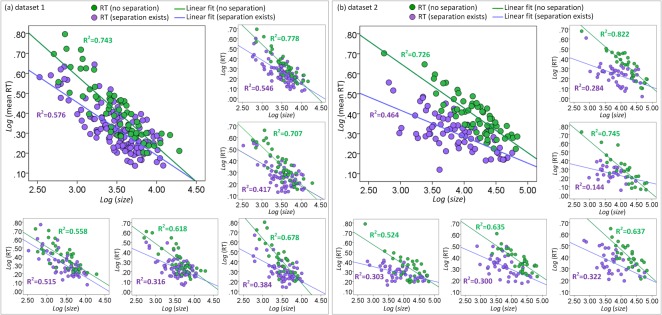
The fitting results of linear functions in two datasets. (a) The fitting solutions for RTs in dataset 1. (b) The fitting solutions for RTs in dataset 2. Dots represent the mean RTs averaged over all observers or RTs of five different observers. Green dots represent the RTs or mean RTs of targets with no separation (*D*_*s*_ = 0). Purple dots represent the RTs or mean RTs of targets with separation (*D*_*s*_ > 0). Curve represents the fitting curve of the linear function.

**Fig 4:**
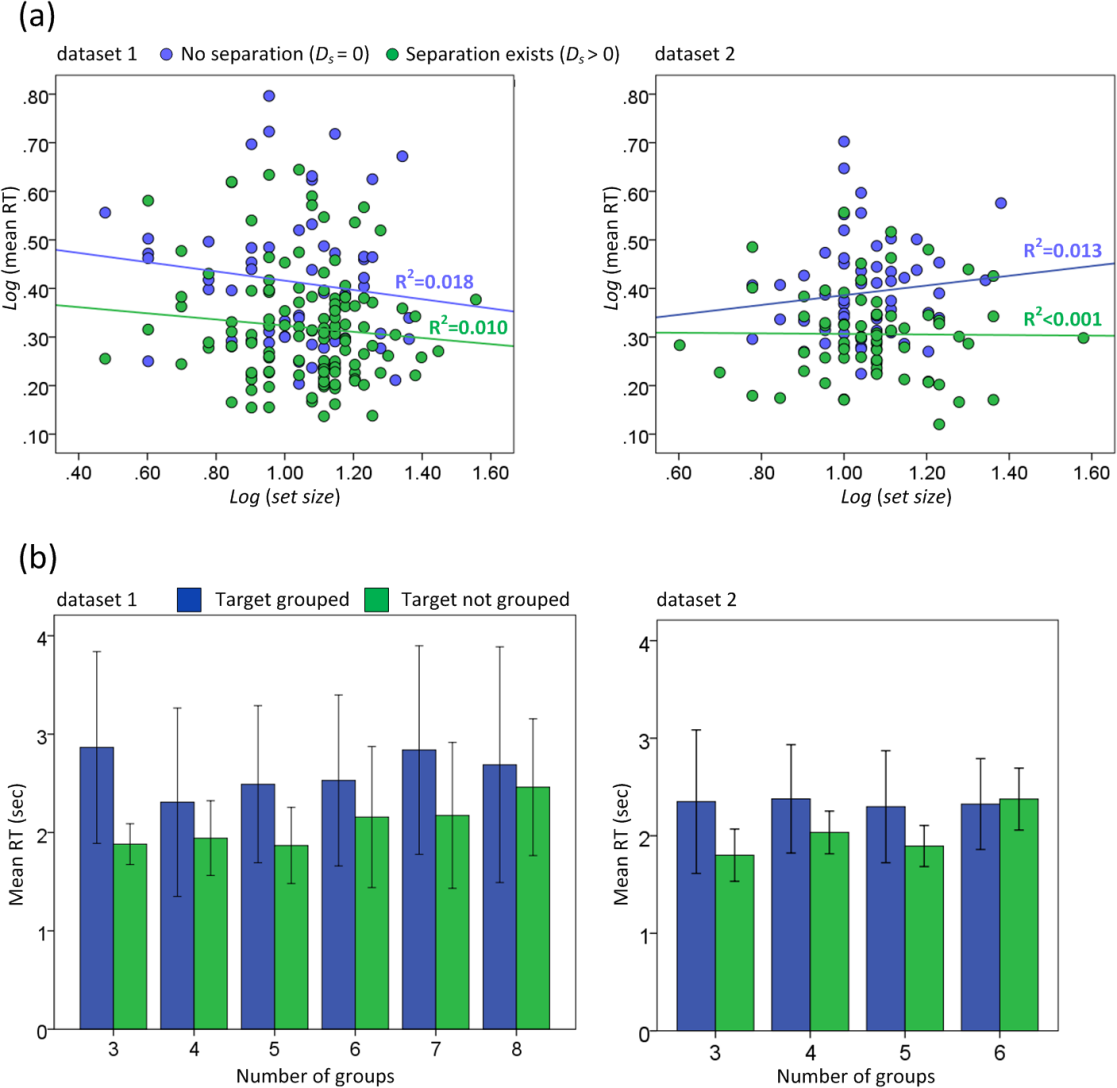
The influence of set size and grouping on search efficiency. (a) Log-transformed mean RT of targets as a function of set size. Blue point is the log-transformed mean RT of a target with no separation (*D*_*s*_ = 0). Green point is the log-transformed mean RT of a target with separation (*D*_*s*_ > 0). (b) Mean RT of targets with different grouping conditions. Error bars represent ± 1 *SDs*.

The experiment investigated the effect of selected factors, e.g., set size, visible size, visual crowding and eccentricity during visual search. In each trial, the observers were required to search for a target in a scene image. The overall mean error rate of all trials with dataset 1 and 2 was 3%. Error trials and outliers (greater than 2.5 standard deviations (*SD*s)) were discarded from further analysis. The mean RT of all trials was 2.36 (*SD* = 0.84) sec in dataset 1 and 2.29 (*SD* = 0.61) sec in dataset 2. Table 1 lists the conditions of the different attributes of the two datasets. An ANCOVA on log-transformed RTs was performed for trials with no separation (i.e., *D*_*s*_ = 0). Log-transformed variables, including *set size*, *size* (or *relative size*), the number of groups and *eccentricity*, were used as continuous independent variables and subject was used as a random categorical variable. Results revealed significant main effects of *size* (*F*(1, 404) = 620.70, *p* < 0.001 for dataset 1; *F*(1, 312) = 413.26, *p* < 0.001 for dataset 2) and *relative size* (*F*(1, 404) = 303.31, *p* < 0.001 for dataset 1; *F*(1, 312) = 244.97, *p* < 0.001 for dataset 2). There was no main effect of *set size* (*F*(1, 404) = 0.96, *p* > 0.33 for dataset 1; *F*(1, 312) = 0.84, *p* > 0.36 for dataset 2), the number of groups (*F*(1, 404) = 0.33, *p* > 0.56 for dataset 1; *F*(1, 312) = 0.09, *p* > 0.77 for dataset 2) and *eccentricity* (*F*(1, 404) = 0.03, *p* > 0.85 for dataset 1; *F*(1, 312) = 3.26, *p* > 0.07 for dataset 2). The interactions among subjects, log-transformed *set size*, *size* (or *relative size*), the number of groups, and *eccentricity* were not significant (*F*s(1, 404) < 1.3, *p* values > 0.23 for dataset 1; *F*s(1, 312) < 0.86, *p* values > 0.35 for dataset 2). These results indicated that visible size, either in its absolute or relative form, played an important role during real-scene visual search when targets were closely flanked by distractors. To further investigate the effect of visible size, the log-transformed mean RT (summed for all subjects) for all targets was calculated as a function of log-transformed *size*. As demonstrated in Fig 3, there was a significant linear relation between log-transformed mean RT and *size* in each datasets. The R^2^ values of the linear function (Eq (2)) were 0.743 and 0.726 (all *p* values < 0.01). The mean errors of this function was 0.060 (*SD* = 0.040) for dataset 1 and 0.047 (*SD* = 0.026) for dataset 2. The linear function was also fitted to the RTs of each subject separately. The R^2^ values of the function ranged from 0.558 to 0.778 for dataset 1 and 0.521 to 0.822 for dataset 2 (all *p* values < 0.01). Although the effect of the RT × Visible Size function was different for different subjects, it generally described a linear downward trend of RTs as visible size increased. Interestingly, the optimized parameter *a* for subjects in dataset 1 (*M* = -0.33, *SD* = 0.06) was significantly lower than the parameter *a* for subjects in dataset 2 (*M* = -0.24, *SD* = 0.04), *F*(1, 27) = 16.40 (*p* < 0.001). The RT decreased more quickly in dataset 1 than in dataset 2 as visible size increased. The log-transformed mean RT of these trials summed for all subjects was also calculated as a function of log-transformed *set size*. As demonstrated in Fig 4A, there was not a clear linear relationship between log-transformed mean RT (blue dots) and *set size*. The R ^2^ values of the two curve fittings were quite small. These results indicate that the variance of other attributes, e.g., visible size or separation, might considerably influence the search efficiency and decrease the effect of set size. These results agree with those of Wolfe et al. [10] that set size is of limited use as a predictor of RT in real scenes.

**Table 1:** Average characteristics of targets and scenes in two datasets. *Size* and *D*_*s*_ are shown in pixels, *eccentricity* is shown in degree (°), SDs are shown in parentheses.

Dataset	*Set size*	Number of groups	*Size*	*Relative size*	*D*_*s*_	*Eccentricity*
1	12.23(5.00)	5.51(1.20)	4102(2893)	0.05(0.03)	118(134)	8.43(3.30)
2	12.18(4.46)	3.82(0.90)	15898(14893)	0.70(0.92)	68(91)	5.81(3.41)

To study the effect of separation on search efficiency, another ANCOVA on log-transformed RTs was performed for trials with separation (*D*_*s*_ > 0). Log-transformed variables, including *set size*, *size* (or *relative size*), *D*_*s*_, the number of groups and *eccentricity*, were used as continuous independent variables and subject was used as a random categorical variable. Results revealed significant main effects of *D*_*s*_ (*F*(1, 885) = 188.94, *p* < 0.001 for dataset 1; *F*(1, 393) = 141.81, *p* < 0.001 for dataset 2). Target-flanker separation played a significant role during real-scene visual search. The main effect of *set size* was only significant in dataset 1(*F*(1, 885) = 8.15, *p* < 0.01) but not significant in dataset 2 (*F*(1, 393) = 0.55, *p* > 0.46). The effect of *set size* on search efficiency seems to be not stable. As shown in Fig 4A, there was not a clear linear relationship between log-transformed mean RT (green dots) and set size. The main effect of *size* (*F*(1, 885) = 645.81, *p* < 0.001 for dataset 1; *F*(1, 393) = 51.62, *p* < 0.001 for dataset 2) and *relative size* (*F*(1, 885) = 328.54, *p* < 0.001 for dataset 1; *F*(1, 393) = 31.22, *p* < 0.001 for dataset 2) were significant, indicating that size factor was also effective for targets with separation (*D*_*s*_ > 0). The main effect of the number of groups was significant as well (*F*(1, 885) = 19.34, *p* < 0.001 for dataset 1; *F*(1, 393) = 5.27, *p* < 0.05 for dataset 2). As demonstrated in Fig 4B, the mean RT generally increased when the group number increased for targets that were not grouped with distractors. However, a similar trend was not observed for targets that were grouped with distractors. If a target is grouped with distractors, the observers are required to search for individual objects in each group, which decreases the effect of grouping. The interactions among subjects, log-transformed *set size*, *size* (or *relative size*), *D*_*s*_, the number of groups, and *eccentricity* were not significant (*F*s(1, 885) < 2.82, *p* values > 0.09 for dataset 1; *F*s(1, 393) < 3.07, *p* values > 0.08 for dataset 2), except for a significant interaction between *size* and *D*_*s*_ (*F*(1, 885) = 183.10, *p* < 0.001 for dataset 1; *F*(1, 393) = 161.38, *p* < 0.001 for dataset 2). This interaction can be explained by the size effect being more pronounced as *D*_*s*_ decreases. As shown in Fig 3, the decreasing speed of green dots (*D*_*s*_ = 0) was generally faster than the decreasing speed of purple dots (*D*_*s*_ > 0). The slope of the fitting lines (i.e. parameter *a* of Eq (2)) for green dots was significantly different from that for purple dots (*F*(1, 27) = 16.36, *p* < 0.001 for dataset 1; *F*(1, 27) = 59.88, *p* < 0.001 for dataset 2). The optimized parameter *a* for targets with no separation (*M* = -0.33, *SD* = 0.06 for dataset 1; *M* = -0.24, *SD* = 0.04 for dataset 2) was smaller than the parameter *a* for targets with separation (*M* = -0.25, *SD* = 0.04 for dataset 1; *M* = -0.12, *SD* = 0.02 for dataset 2). The size factor is more effective when *D*_*s*_ becomes smaller.

**Fig 5:**
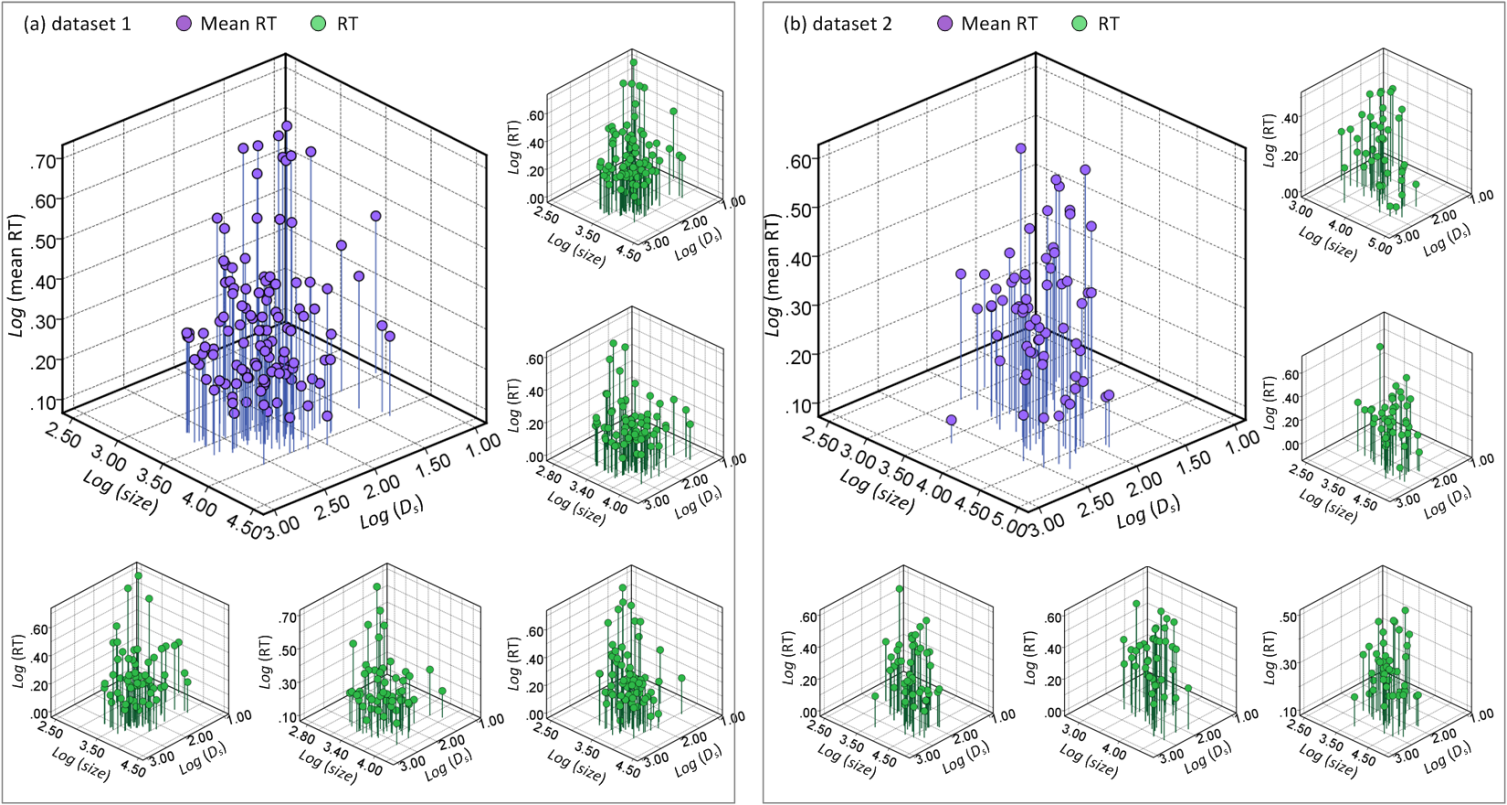
RT as a function of *size* and *D*_*s*_ in two datasets when *D*_*s*_ is greater than 0. (a) The distribution pattern of RTs in dataset 1. (b) The distribution pattern of RTs in dataset 2. Purple dots represent the mean RTs average over all observers. Green dots represent the RTs of five different observers. Blue or green line represents the RT of a dot.

To quantitatively study the effect of separation, targets (*D*_*s*_ > 0) were selected from the two datasets. The mean RT for these targets was calculated as a function of *size* and *D*_*s*_. As demonstrated in Fig 5, the mean RT decreased gradually as *size* or *D*_*s*_ increased. Targets with a large *size* and *D*_*s*_ resulted in fast RTs. The RT × Visible Size × Separation function (Eq (3)) was fitted to the mean RTs for these targets. The R^2^ values of the fittings were 0.743 (dataset 1) and 0.740 (dataset 2), respectively (all *p* values < 0.01). The mean errors of the two fitting solutions were 0.045 (*SD* = 0.038) and 0.039 (*SD* = 0.028). To further test the effect of the RT × Visible Size × Separation function, this function (Eq (3)) was fitted separately to the search results of each subject. Fig 5 presented five examples of the fitting results in either dataset. The R^2^ values of these fittings ranged from 0.524 to 0.723 for dataset 1 and from 0.526 to 0.706 for dataset 2 (all *p* values < 0.01). There was a statistical difference between Eqs (2) and (3) in mean error of each fitting solution (*F*(1, 27) = 12.29, *p* < 0.01 for dataset 1; *F*(1, 27) = 6.22, *p* < 0.05 for dataset 2). The mean error of each fitting solution with Eq (3) (*M* = 0.055, *SD* = 0.007 for dataset 1; *M* = 0.049, *SD* = 0.009 for dataset 2) was significantly lower than that with Eq (2) (*M* = 0.064, *SD* = 0.007 for dataset 1; *M* = 0.060, *SD* = 0.012 for dataset 2). This result indicated that search efficiency can be better explained by the combination of visible size and separation than by visible size alone. Interestingly, the optimized parameter *a* of Eq (3) for subjects in dataset 1 (*M* = -0.21, *SD* = 0.03) was significantly lower than that for subjects in dataset 2 (*M* = -0.06, *SD* = 0.03), *F*(1, 27) = 154.89 (*p* < 0.001). The optimized parameter *b* for subjects in dataset 1 (*M* = -0.11, *SD* = 0.02) was significantly higher than that for subjects in dataset 2 (*M* = -0.15, *SD* = 0.03), *F*(1, 27) = 13.44 (*p* < 0.01). It seems that the effect of visible size on search efficiency was greater when the visible size was relatively small (e.g., in dataset 1). The effect of separation was greater when the visible size was large enough to provide sufficient visual information about the target. We attempted to include the variable *eccentricity* in the function because the main effect of *eccentricity* was also significant (*F*(1, 885) = 25.64, *p* < 0.001 for dataset 1; *F*(1, 393) = 10.02, *p* < 0.01 for dataset 2). Eq (4) was fitted to the mean RTs for these targets. The R^2^ values of the fittings were 0.768 (dataset 1) and 0.764 (dataset 2), respectively (all *p* values < 0.01). The mean errors of the two solutions were 0.042 (*SD* = 0.034) and 0.035 (*SD* = 0.026). When Eq (4) was fitted separately to the search results of each subject, the R^2^ values of the fittings ranged from 0.527 to 0.759 for dataset 1 and 0.537 to 0.727 for dataset 2 (all *p* values < 0.01). The mean error of each fitting solution with Eq (4) (*M* = 0.054, *SD* = 0.007 for dataset 1; *M* = 0.047, *SD* = 0.009 for dataset 2) was lower than that with Eq (3) (*M* = 0.055, *SD* = 0.007 for dataset 1; *M* = 0.049, *SD* = 0.009 for dataset 2), but the difference was not statistically significant (*F*(1, 27) = 0.11, *p* > 0.7 for dataset 1; *F*(1, 27) = 0.15, *p* > 0.6 for dataset 2). The performance of the RT × Visible Size × Separation function was slightly improved when *eccentricity* was included in the function.

## Discussion

In this study, we proposed a computational model of search efficiency in real scenes. We determined that the RT × Set Size function, the standard measure of efficiency, was less effective for measuring search efficiency in real scenes than in artificial scenes. Compared with artificial scenes, real scenes are more complex and meaningful [12]. The relatively inefficiency of the RT × Set Size function may be caused by the variance of various factors in complex scenes. We quantitatively investigated the effect of selected factors, e.g., set size, visible size, visual crowding and eccentricity, on search efficiency. Classical studies of visual attention have demonstrated that these factors play important roles during searches of artificial scenes. However, less is known about the contribution of these factors to the search efficiency of real scenes, in which the distractor set is highly heterogeneous. We determined that visible size and target-flanker separation significantly accelerated the visual search when pictures of target objects were used as target templates. The experimental results from the two datasets indicated that the RT × Visible size × Separation function could be a relatively rough measurement function for search efficiency in real scenes. These findings have implications for search efficiency modeling and may contribute to understanding the mechanisms of real-world visual search.

The influence of high-level features should be considered when constructing computational search efficiency models. Studies of eye movements have demonstrated that high-level features, such as context and target template, guide the deployment of attention and facilitate the visual search in real scenes [2,40,42,47]. These high-level features, together with low-level features, should be considered in the approach to measuring search efficiency. For example, Ehinger et al. [2] proposed a computational search guidance model by studying the eye movements of observers during a real-scene visual search task. This model predicted approximately 94% human agreement, and the scene context provided the most accurate guidance information. Torralba et al. [40] presented a contextual guidance model of attention that combines bottom-up saliency, scene context and top-down factors, which could predict the regions in real scenes that are likely to be fixated upon by human observers. Compared with previous studies, this study investigated the extent to which each of the selected factors explains the efficiency of real-scene search and how to integrate these factors to better explain search efficiency. Our findings extend classical visual search theories to real-scene visual search. In addition to the factors investigated in this study, findings from multiple academic field of study, such as scene perception and object detection, can also be considered when studying the mechanisms of real-world visual search.

One difficulty with applying the RT × Set Size function to more complex real scenes is counting the set size in real scenes. Wolfe et al. [10] used the number of labeled regions as a surrogate for set size and found that search was efficient in real scenes. However, the experiments performed by Wolfe et al. also indicated that the number of labeled items in a scene was a relatively poor predictor of search efficiency. In accordance with this finding, we found that the variance of various features (other than set size) decreased the effect of the RT × Set Size function. Interestingly, we also found that grouping might be correlated with how set size is counted. Both MRI (fMRI) and behavioral studies have revealed that if distractors can be grouped based on physical similarity, geometric relationships, or regular configurations, the grouped distractors can be rejected simultaneously to facilitate target detection [7,48,49]. The RT × Set Size function may be improved by treating the grouped distractors as an item in the set size. Therefore, it is important to study suitable grouping principles for real-scene visual search. In this study, it was more suitable to include the effect of grouping in the set size when a target was not grouped with distractors. If the target was in a group, the observers were required to search for the target item-by-item, which decreased the grouping effect. This finding could contribute to including the grouping effect in the definition of set size in real scenes. However, more studies are needed to determine grouping principles to define the set size for real-scene search tasks.

The search efficiency for a real object strongly depends on the object’s visible size, which in this study was correlated with the amount of information about an object in real scenes. A larger visible size, either in an absolute or relative form, could provide richer visual information about an object facilitating object specification in a real scene. This finding agrees with those of previous studies: a larger size leads to shorter RTs in artificial [4,50] or real scenes [10]. The size factor is more effective for measuring search efficiency in real scenes than set size. A significant linear relationship between log-transformed RT and visible size was observed, which was somewhat similar to the rough linear relationship observed between RT and the square root of size [10]. The difference is that the largest sizes were removed from the analysis by Wolfe et al. because the number of observations for large stimuli was relatively small in their experiment. In contrast, we tested the effect of a large size on search efficiency, with the visible size of the targets ranging from 336 to 19295 pixels in the datasets. We determined that the visible size was more effective for measuring search efficiency when it was relatively small. The acceleration effect of visible size on RT decreased gradually after the object was large enough to provide sufficient information about its appearance.

Previous studies have demonstrated that visual crowding has a deleterious effect on visual performance during search tasks such as letter identification and vernier acuity. As an extension of these studies, we defined a descriptor of target-flanker separation, i.e., *D*_*s*_, to test the effect of crowding in real scenes. We determined that the RT decreased significantly as *D*_*s*_ increased. This result indicates that visual crowding also has a negative effect on search efficiency in real scenes. Moreover, target-flanker separation not only increases performance accuracy [22] but also accelerates visual search in real scenes. Similar to the visible size factor, the acceleration effect of separation on RT decreases gradually as separation increases continuously. These findings are different from the results reported by Wolfe et al. (who reported no reliable difference between crowded and uncrowded displays of real scenes) [10]. The reason is that the “crowded” stimuli in Wolfe’s study were well specified and separated in indoor scenes. However, many real objects are not visually unique and commonly share their contours with each other. The deleterious effect of visual crowding increases with increasing similarity between the target and distractors [18,33].

The RT × Set size function is a relatively poor predictor of RT in real scenes [10]. Based on a combination of low-level and high-level features, we propose an RT × Visible size × Separation function (Eqs (2), (3) and (4)) to measure search efficiency for real objects in real scenes. The results indicate that this function can be used to roughly measure the RT for real objects. The function also describes the effect of visible size and separation on the search efficiency. The constants in this function are determined by the variance of the two factors. The performance of this function was further improved when eccentricity was added to the function as a variable. This study mainly focused on urban scenes, leaving other, more natural, real scenes (e.g., forests) unaddressed. The scene context of natural scenes, such as spatial knowledge or object categories, is different from that of urban scenes. It will be necessary to refine the proposed function when it is applied to other scenes, a process that will be considered in future work. It would also be interesting to include the improved set size factor in the proposed function for better measurement performance.
